# Dose-Dependent Effect of Mesenchymal Stromal Cell Recruiting Chemokine CCL25 on Porcine Tissue-Engineered Healthy and Osteoarthritic Cartilage

**DOI:** 10.3390/ijms20010052

**Published:** 2018-12-23

**Authors:** Luise Lüderitz, Tilo Dehne, Michael Sittinger, Jochen Ringe

**Affiliations:** Charité-Universitätsmedizin Berlin, Corporate Member of Freie Universität Berlin, Humboldt-Universität zu Berlin and Berlin Institute of Health, Tissue Engineering Laboratory, Berlin-Brandenburg Center for Regenerative Therapies and Department of Rheumatology and Clinical Immunology, 10117 Berlin, Germany; luise.luederitz@charite.de (L.L.); tilo.dehne@charite.de (T.D.); michael.sittinger@charite.de (M.S.)

**Keywords:** CCL25, Thymus-expressed chemokine, cartilage, in vitro osteoarthritis model, in situ tissue engineering

## Abstract

Thymus-expressed chemokine (CCL25) is a potent cell attractant for mesenchymal stromal cells, and therefore it is a candidate for in situ cartilage repair approaches focusing on the recruitment of endogenous repair cells. However, the influence of CCL25 on cartilage is unknown. Accordingly, in this study, we investigated the effect of CCL25 on tissue-engineered healthy and osteoarthritic cartilage. Porcine chondrocytes were cultured in a three-dimensional (3D) micromass model that has been proven to mimic key-aspects of human cartilage and osteoarthritic alterations upon stimulation with tumor necrosis factor-α (TNF-α). Micromass cultures were stimulated with CCL25 (0, 0.05, 0.5, 5, 50, 500 nmol/L) alone or in combination with 0.6 nmol/L TNF-α for seven days. Effects were evaluated by life/dead staining, safranin O staining, histomorphometrical analysis of glycosaminoglycans (GAGs), *collagen type II* (*COL2A1*) real-time RT-PCR and Porcine Genome Array analysis. 500 nmol/L CCL25 led to a significant reduction of GAGs and *COL2A1* expression and induced the expression of matrix *metallopeptidases* (*MMP*) *1*, *MMP3*, *early growth response protein 1* (*EGR1*), and *superoxide dismutase 2* (*SOD2*). In concentrations lower than 500 nmol/L, CCL25 seems to be a candidate for in situ cartilage repair therapy approaches.

## 1. Introduction

Regenerative cell-based therapies for osteoarthritis (OA), like autologous chondrocyte implantation, have already reached clinical practice for the treatment of traumatic focal and early degenerative cartilage defects [1]. Furthermore, therapy approaches that combine microfracture and cell-free implants have also reached the clinic [2]. In such in situ tissue engineering applications, blood and bone marrow cells fill the defect and induce, supported by implants, the formation of cartilage [3]. This strategy avoids cell harvesting and expansion, donor site morbidity-related pain and impairment, and reduces the complexity of surgical interventions. Next generation in situ therapy approaches focus on molecules that actively recruit endogenous repair cells, such as mesenchymal stem- or stromal cells (MSC) [3].

MSC play a pivotal role in the development of regenerative OA therapies. They have been found in distinct joint locations, like synovial membrane and fluid, infrapatellar fat pad, and bone marrow [4,5]. MSC differentiate into chondrocytes, secrete regenerative factors, exosomes and microparticles, and modulate immune responses [6,7,8]. Different molecules, such as growth factors and chemokines, have been identified to mediate MSC migration in vitro [9]. MSC express several chemokine receptors [10], and chemokines, like stromal cell-derived factor 1α (CXCL12), CCL2-4, and -7, are known to be expressed at damaged tissue sites and mediate MSC homing, leading to microenvironment changes and functional improvement [11,12,13].

In a recent study, thymus-expressed chemokine (CCL25) was pointed out to be a candidate for in situ tissue engineering [14]. Its chemotactic effect on human bone marrow MSC was shown in Boyden chamber assays, and expression profiling of CCL25 stimulated MSC revealed the induction of genes coding for factors that are involved in the migration and homing of bone marrow cells [15]. CCL25 is known to be a ligand of the C-C motif chemokine receptor 9 (CCR9) on T cells and different other cells that are located in the small intestine [16]. It plays an important role for T cell trafficking in thymus and small intestine [17,18,19]. CCL25 is also present in the synovial fluid of healthy people and patients with OA or rheumatoid arthritis [20].

Our overall aim is to establish an in situ cartilage repair approach based on injectable hydrogel hyaluronic acid combined with CCL25 to recruit repair cells to damaged or degenerated cartilage sites [21]. However, the role of CCL25 in the joint cavity and its influence on cartilage remains unclear.

Therefore, in this study, we investigated the effect of recombinant human CCL25 on migration of porcine MSC in a Boyden chamber assay, and on tissue-engineered cartilage, as depicted in the experimental flow chart (Figure 6). For cartilage formation, porcine chondrocytes were three-dimensional (3D)-cultured for 14 days in high-density micromass cultures to form extracellular matrix (ECM), and stimulated for further seven days with different concentrations of CCL25 alone or in combination with tumor necrosis factor α (TNF-α). The porcine micromass model is reproducible, well characterized, and has been proven to mimic substantial aspects of human native cartilage and OA alterations upon TNF-α stimulation [22]. For the first time, we observed a dose range of CCL25 without negative effects on tissue-engineered cartilage ECM and chondrocyte viability, which is relevant to establish an in situ repair approach with CCL25 to recruit cells to damaged or degenerated cartilage sites.

## 2. Results

### 2.1. CCL25 Induced Migration of Porcine MSC

**Figure 1:**
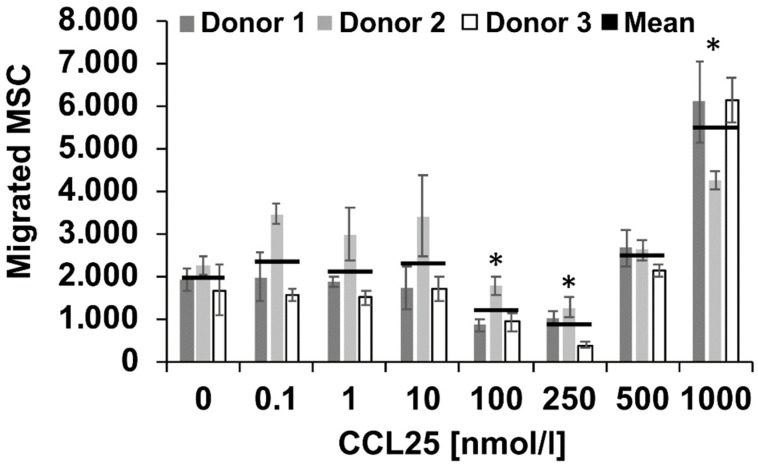
Mesenchymal stem- or stromal cells (MSC) migration assay. 96-well format chemotaxis assays were performed to investigate the dose-dependent migratory effect of recombinant human thymus-expressed chemokine (CCL25) on porcine MSC (*n* = 3 donors, all measured in triplicates). Recombinant human CCL25 induces the migration of porcine MSC. The results demonstrate a biological effect of human CCL25 on pig cells comparable to human cells [15]. Data are expressed as mean numbers of migrated MSC ± standard deviation (SD). * indicates *p* < 0.05 compared with 0 nmol/L CCL25 control.

To assess whether recombinant human CCL25 activates porcine cells, we performed a migration assay with porcine MSC. Cells of all three donors showed a dose-dependent migratory response (Figure 1). In the CCL25-free control group, 1964 ± 300 (mean cell number ± standard deviation SD) out of 30,000 MSC passed the membrane. We measured significant MSC migration at 1000 nmol/L CCL25 (5503 ± 1084 cells). Lower concentrations did not stimulate MSC migration. These results are comparable to human MSC [15] and they demonstrate that recombinant human CCL25 shows functional activity on porcine cells.

### 2.2. Effect of CCL25 on Chondrocyte Viability

**Figure 2:**
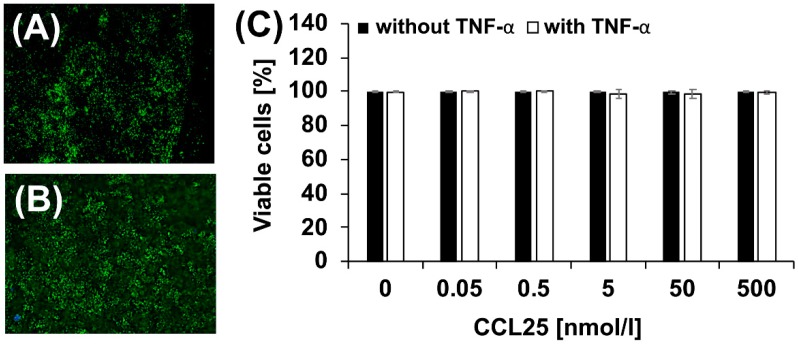
Life/dead assay. (**A**) Micromass after treatment with 500 nmol/L CCL25, most chondrocytes were viable (green, fluorescein diacetate stained), only a few were dead (red, propidium iodide stained). (**B**) Combination of 500 nmol/L CCL25 with 0.6 nmol/L tumor necrosis factor α (TNF-α) did not result in significant increased number of dead chondrocytes. (magnification of all images ×40) (**C**) Micromasses of each donor group (*n* = 4 donor groups) stimulated with CCL25 (0, 0.05, 0.5, 5, 50, 500 nmol/L) without (black) or with 0.6 nmol/L TNF-α (white) for seven days. The diagram shows the mean numbers of viable chondrocytes ± SD. CCL25 has no significant effect on chondrocyte viability.

Next, we stimulated untreated and TNF-α treated porcine chondrocytes in 3D high-density micromasses with different CCL25 concentrations for seven days (Figure 2). Live/dead staining revealed a high cell viability of 98–100% even at the highest concentration of 500 nmol/L CCL25 (Figure 2A,C) and regardless of the combination with TNF-α (Figure 2B,C). All in all, no significant or dose-dependent stimulation of cell death was observed.

### 2.3. CCL25-dose Dependent Reduction of GAGs

**Figure 3:**
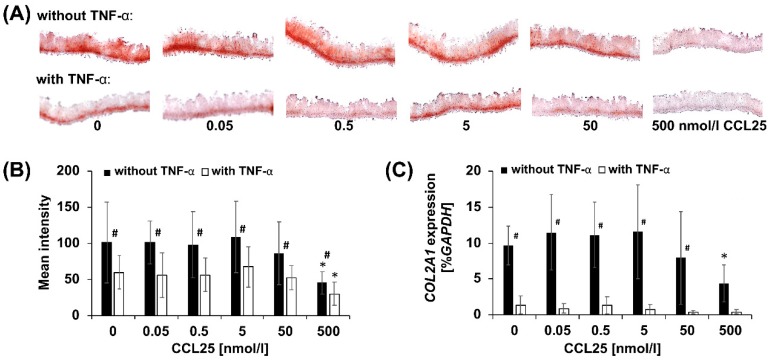
Histomorphometrical analysis and real-time RT-PCR. (**A**) Safranin O staining of representative stained slices to visualize the negative correlation between cartilage glycosaminoglycan content and CCL25 concentration in porcine micromass cultures with and without TNF-α (magnification ×40). (**B**) Semi-quantitative histomorphometric analysis of safranin O stained micromasses of all four donor groups after seven days of CCL25 treatment without or with 0.6 nmol/L TNF-α. Data are expressed as mean intensities ± SD. (**C**) Semi-quantitative RT-PCR of micromasses of all four donor groups after seven days of CCL25 treatment without or with 0.6 nmol/L TNF-α supplementation. Data are expressed as mean expression values in percent *GAPDH* ± SD. * indicates *p* < 0.05 for micromasses induced with CCL25 as compared with 0 nmol/L control. # indicates *p* < 0.05 for micromasses induced with CCL25 compared with micromasses induced with CCL25 and TNF-α.

Safranin O staining of tissue-engineered, not TNF-α treated micromasses revealed a negligible effect of CCL25 on GAG-content up to a concentration of 5-50 nmol/L (Figure 3A). At higher concentrations, GAG-content was decreased. This was confirmed by semi-quantitative histomorphometry (Figure 3B). The mean intensity and SD of unstimulated controls was 101.0 ± 64.1. ANOVA indicated significant differences between groups, post hoc analysis demonstrated no significant differences between controls and cultures stimulated with 0.05, 0.5, 5, and 50 nmol/L CCL25. At 500 nmol/L mean intensities significantly decreased to 45.1 ± 18.0. Therefore, a dose-dependent effect on GAG-content was observed. In presence of TNF-α, safranin O staining indicated a decreased GAG-content at 500 nmol/L CCL25 (Figure 3A). Histomorphometry confirmed a significant decrease in mean intensity from 69.7 ± 27.0 at 0 to 29.9 ± 18.1 at 500 nmol/L CCL25 (Figure 3B). Independent of the CCL25 concentration, GAG-content was significantly lower in TNF-α treated as compared to untreated micromasses (Figure 3B).

### 2.4. CCL25-Dependent COL2A1 Expression

The decrease in GAG-content in micromass cultures without TNF-α runs parallel to reduced *COL2A1* expression. Real-time RT-PCR revealed that *COL2A1* levels of CCL25-free controls (9.6 ± 2.8% expression of housekeeping gene *GAPDH*) remained approximately stable up to a concentration of 5 nmol/L CCL25 (Figure 3C). Subsequently, expression decreased slightly at 50 and significantly (4.4 ± 2.7% *GAPDH*) at 500 nmol/L CCL25. Stimulation with TNF-α resulted in a low *COL2A1* expression of 1.3 ± 1.2% *GAPDH* in control cultures, and no significant changes up to 500 nmol/L CCL25 (Figure 3C). Up to 50 nmol/L CCL25, the *COL2A1* expression levels were significantly lower in TNF-α treated as compared to untreated micromasses (Figure 3C).

### 2.5. Gene Expression Profiling of Untreated and TNF-α Treated Micromasses

**Figure 4:**
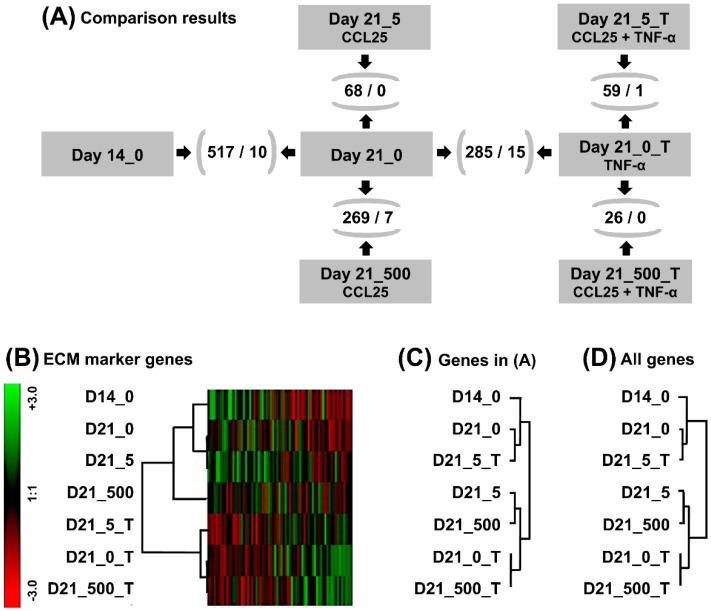
CCL25-induced changes in the gene expression profile of tissue-engineered cartilage and hierarchical cluster analysis. (**A**) RNA from chondrocyte micromass cultures of all four donor groups was pooled equally for the treatment groups: day 14 without CCL25 stimulation (Day 14_0), day 21 without CCL25 (Day 21_0) or with 5 nmol/L CCL25 (Day 21_5) or 500 nmol/L CCL25 (Day 21_500) stimulation for seven days, and day 21 without CCL25 plus 0.6 nmol/L TNF-α (Day 21_0_T) or with 5 nmol/L CCL25 (Day 21_5_T) or 500 nmol/L CCL25 (Day 21_500_T) stimulation for seven days, and gene expression profiles were generated with Porcine Genome arrays (Figure 6). These groups were compared with each other (black arrows) using Affymetrix GCOS software. Number in parenthesis represent differentially expressed genes between two groups and the subset of genes (/) that is known in context of cartilage. Hierarchical cluster analysis was performed for (**B**) 69 cartilage genes listed in Supplementary Table S1, (**C**) 1044 genes that were differentially expresses at least between two groups (see A), and 14,785 genes with known Gene symbol on porcine GeneChip. Red: down-regulation, green: up-regulation.

To analyze the changes on gene expression level in more detail, gene expression profiling with Porcine Genome Arrays was conducted (Figure 4). After applying our selection criteria, the expression of 787 probe-sets was significantly changed. Excluding non-assignable probe-set IDs and double entries, 517 genes were differentially expressed; unraveling the culture effect (Figure 4A). At first, the quality of tissue-engineered cartilage (non-stimulated micromasses at day 14 and 21) that we evaluated based on cartilage markers reviewed by J. Martel-Pelletier (Supplementary Table S1) [23]. Genes coding for cartilage collagens (*COL2A1, -6A1, -6A3, -9A1, -9A2, -11A1*) and proteoglycans (*aggrecan*; *ACAN*, *biglycan*; *BGN, decorin*; *DCN*, *fibromodulin*; *FMOD*, *link protein*; *HAPLN1*, *lumican*; *LUM*; *versican*; *VCAN*) were detected. None of them was differentially expressed between day 14 and 21 (Supplementary Table S1). Marker genes known in context of differentiation and hypertrophy were not detected or low expressed (*COL1A1*). *COL10A1* was present on day 14 but not 21. Expression of catabolic factor genes, like *matrix metallopeptidases* (*MMP1-3, -13, -14*) was measured; *MMP3* and *-13* were down-regulated at day 21. All of these data implied appropriate quality of tissue-engineered cartilage for CCL25 testing.

Next, day 14 micromasses were stimulated with TNF-α for seven days were analyzed. When comparing with unstimulated day 21 micromasses, 285 genes, including 15 cartilage genes, were differentially expressed (Figure 4A). Expression of ECM genes (*ACAN*, *chondroitin sulfate proteoglycan 4*; *CSPG4*, *COL1A1, -2A1, -9A1, -A2, -11A1, cartilage oligomeric matrix protein; COMP, HAPLN1*) was down-, and of catabolism (*jun B proto-oncogene; JUNB, MMP1, -3, -13*) and inflammation related genes (*interleukin 18; IL18, prostaglandin E synthase; PTGES*) was up-regulated. These data show relevance of the porcine model to simulate OA-like alterations and are in line with safranin O staining and PCR results (Figure 3A–C).

### 2.6. Comparative Expression Profiling of CCL25 Treated Micromasses

To study the effects of CCL25 on tissue-engineered cartilage, micromasses stimulated for seven days with 5 and 500 nmol/L CCL25 were compared with non-stimulated (0 nmol/L CCL25) micromasses. We found 68 differentially expressed genes in the 5 and 269 in the 500 nmol/L group (Figure 4A). No cartilage marker gene was significantly up-or down-regulated in the 5 nmol/L and seven in the 500 nmol/L group (Supplementary Tables S1 and S2). Expression of transcription factor *JUNB, MMP1*, and *-13* were up-, and of *COL1A1, -2A1, -6A1,* and *COMP* down-regulated. Decreased *COL2A1* expression was also seen in RT-PCR (Figure 3C), and the increased expression of *MMP1*, and *-13* was consistent with the decreased GAG-content in micromasses treated with 500 nmol/L CCL25 (Figure 3A,B).

In the 5 nmol/L group, hyaluronan mediated motility receptor (HMMR), cell cycle regulator cyclin A2 (CCNA2) and actin-bundling protein coding espin (ESPN) were most down-regulated (Supplementary Table S2). Involucrin (IVL), solute carrier family 12 member 1 (SLC12A1), and tight-junction component MARVEL domain-containing protein 3 (MARVELD3), so far all not discussed in the context of cartilage or CCL25, were most up-regulated. Stimulation with 500 nmol/L CCL25 resulted in a four-fold higher number of differentially expressed genes (Supplementary Table S2). We found increased expression levels of early growth response protein 1 (EGR1) and superoxide dismutase 2 (SOD2); both being capable to suppress COL2A1 expression. Calsequestrin 2 (CASQ2) and sphingomyelin phosphodiesterase 1 (SMPD1) were most down-, whereas tetratricopeptide repeat domain 28 (TTC28), ankyrin family member ankyrin 3 (ANK3), and CXCL2 were most up-regulated. Among these genes, CXCL2 was already discussed in the context of cartilage.

### 2.7. GeneChip Analysis of CCL25 Treated TNF-α Stimulated Micromasses

TNF-α micromasses treated for seven days with 5 and 500 nmol/L CCL25 were compared with non-stimulated micromasses. 59 genes were differentially expressed in the 5 and 26 in the 500 nmol/L CCL25 group (Figure 4A). With *fibronectin* (*FN1*), one cartilage marker gene was significantly regulated (down) in the 5 nmol/L group, whereas no marker gene was differentially expressed in the 500 nmol/L group (Supplementary Tables S1 and S3). This was consistent with mean safranin O intensities and RT-PCR values (Figure 3B,C). In the 5 nmol/L group, *glutamate decarboxylase 2* (*GAD2*) and *interleukin-8* (*IL8*) were most down- and *FosB proto-oncogene, AP-1 transcription factor subunit* (*FOSB*) most up-regulated. *IL8* was also strong down-regulated in the 500 nmol/L group (Supplementary Table S3). The expression of *BSCL2* coding multi-pass transmembrane protein seipin was most down-regulated. To sum up, treatment with 5 or 500 nmol/L CCL25 resulted in a very low number of significantly regulated genes, indicating a strong TNF-α effect in addition to the CCL25 effect.

### 2.8. Hierarchical Clustering and Functional Classification of Significantly Regulated Genes

Hierarchical clustering of cartilage marker genes (Supplementary Table S1) revealed three main groups (Figure 4B). The first group encompassed untreated and low dose treated (5 nmol/L CCL25) chondrocyte micromasses, the second group high dose treated (500 nmol/L CCL25) micromasses, and the third group all TNF-α stimulated micromasses; no matter if CCL25 treated or not. Furthermore, clustering of 1044 genes differentially expressed in at least one of the six group comparisons resulted in two main groups with two subgroups, respectively (Figure 4C). Interestingly, untreated day 21 micromasses clustered with TNF-α and 5 nmol/L CCL25 treated micromasses. We made the same observation when clustering all 14,785 genes with known gene symbol (Figure 4D); maybe indicating an inhibitory effect of low CCL25 doses on TNF-α signaling.

**Figure 5:**
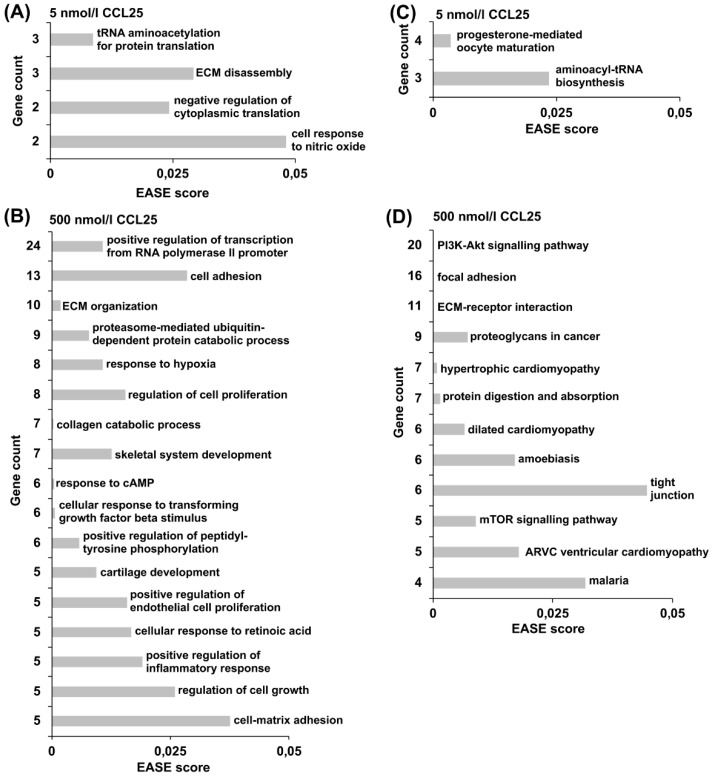
Assignment of differentially expressed genes to Gene ontology (GO) terms in the biological process (BP) domain and Kyoto Encyclopedia of Genes and Genomes database (KEGG) pathways. Genes that were differentially expressed in chondrocyte micromass cultures from all four donor groups after stimulation with (**A**,**C**) 5 nmol/L CCL25 and (**B**,**D**) 500 nmol/L CCL25 were analyzed with the DAVID Functional Annotation Tool. Functional annotation charts were generated using the DAVID categories (**A**,**B**) GO terms in the BP domain and (**C**,**D**) KEGG pathway analyses. The number of gene count per significantly enriched GO term or KEGG pathway is plotted over the *p*-value (EASE score). *P*-values < 0.05 were considered significant.

Because of the strong TNF-α effect, subsequent Gene ontology (GO) terms analysis and Kyoto Encyclopedia of Genes and Genomes database (KEGG) pathway analysis was exclusively performed for unstimulated micromasses. Analysis of genes that were differentially expressed after treatment with 5 nmol/L CCL25 revealed that the most highly enriched GO terms included protein translation, ECM breakdown, and cellular response to nitric oxide (Figure 5A). 500 nmol/L CCL25 led to a higher number of 33 significantly enriched GO terms (Figure 5B). Considering terms matching with five or more genes, several of these genes are involved in collagen catabolic processes, cartilage development and ECM organization. KEGG pathway analysis revealed no significantly enriched relevant pathway in the 5 nmol/L group (Figure 5C). With 20 differentially expressed genes, the PI3K-Akt signaling pathway was the dominant pathway that was influenced by 500 nmol/L CCL25 (Figure 5D). Genes of this pathway overlap with two other significantly enriched networks: focal adhesion pathway and ECM-receptor pathway.

## 3. Discussion

In this study, we investigated dose-dependent effects of CCL25 on porcine MSC migration, and on healthy and OA-like cartilage formed by porcine chondrocytes. So far, being only known for human MSC [15], we report a comparable chemotactic effect of CCL25 on porcine MSC in vitro. This finding is of highly relevance for future in vitro and in vivo model development within the scope of in situ tissue engineering. Furthermore, for the first time, we report effects of CCL25 on chondrocyte viability and cartilage. CCL25 in five different concentrations alone (0.05, 0.5, 5, 50, 500 nmol/L) or in combination with TNF-α to simulate key-aspects of OA were tested for seven days on 14 days old high-density chondrocyte micromass cultures [22], which did not result in a significant stimulation of cell death. In contrast, semi-quantitative histomorphometric analysis revealed a significant dose-dependent reduction of GAG-content and RT-PCR of *COL2A1* expression at 500 nmol/L CL25. For lower doses, we measured stable GAG-content and *COL2A1* expression. When TNF-α was added, the graduation of results was limited due to its overlapping effects.

To further examine the underlying reasons for ECM alterations that were seen when tissue-engineered cartilage was induced with high CCL25 concentrations, we compared the gene expression profiles of micromasses induced with 5 and 500 nmol/L CCL25. We found that the expression of *EGR1*, coding for a nuclear transcription factor, was 1.87-fold increased upon stimulation with 500 nmol/L CCL25. Up-regulated expression of *EGR1* was able to suppress *COL2A1* transcription in chondrocytes [24,25]. We also found that *SOD2* expression was 2.64-fold increased after treatment with 500 nmol/L CCL25. *SOD2* is located in mitochondria and catalyzes superoxide, one of the main reactive oxygen species (ROS) produced by chondrocytes, into hydrogen peroxide [26]. Several proinflammatory cytokines, such as IL-1, -4, and -6, and TNF-α were pointed out to induce *SOD2* expression [26,27]. Therefore, CCL25 seems to be another *SOD2* inducing cytokine. ROS are known to take part in intracellular signaling pathways and transcription factor regulation [28]. One of the redox-sensitive transcription factors is EGR1 [29,30]. A previous study observed that higher hydrogen peroxide levels led to an induced *EGR1* transcription [31]. Furthermore, we observed that the expression of *MMP1* and *MMP13* was elevated 2.00-fold. Studies postulated that *MMP1* expression is SOD2 dependent and it can be reversed by catalase co-expression [32,33,34]. In addition, *SOD2* overexpression enhances *MMP1* promoter activity through pathways, including transcription factors like ETS proto-oncogene 1 (ETS1), jun proto-oncogene, AP-1 transcription factor unit (JUN), and mitogen-activated protein kinases (MAPK1, -3, -8) [26,32]. The *MMP1* and *MMP13* promotors both contain JUN binding sites [35,36].

Micromass cultures stimulated with 500 nmol/L CCL25 up-regulated *periostin* (*POSTN*) expression. This secreted extracellular protein interacts with MSC during tissue repair and takes part in collagen cross-linking [37]. *POSTN* expression is transcriptionally regulated by mechanical stress, growth factors, hormones and cytokines, and it is up-regulated in OA cartilage [38,39]. Previous studies observed that periostin induces MMP13 secretion in a dose- and time-dependent manner and down-regulates *COL2A1* expression [40]. We also found a 2.46-fold increase of *janus kinase 2* (*JAK2*). JAK2 is associated with cytokine receptor signaling pathways, and JAK2 inhibition can reduce *MMP13* induction in chondrocytes [41,42]. Both MMP1 and MMP13 are capable of cleaving collagen type II and are key-players in OA and RA. Taken together, we assume that high concentrations of CCL25 lead to ECM breakdown via superoxide dismutase and periostin induced MMP expression in our micromass model.

We also detected *leprecan-like 4* (*LEPREL4*), a gene involved in collagen biosynthesis, to be down-regulated at 500 nmol/L CCL25 (-1.62-fold) [43]. COL10A1 is described as a marker for chondrocyte hypertrophy that is increased in cartilage during later OA stages [44,45]. The expression level also correlates with cartilage mineralization [46]. This cartilage marker was not significantly up- or downregulated in any of the compared treatment groups. Moreover, the expression of *CXCL2* was 4.59-fold increased in 500 nmol/L CCL25 treated micromasses. It is known that proinflammatory cytokines, like IL-1β and TNF-α, induce *CXCL2* expression in chondrocytes and that *CXCL2* is up-regulated in OA cartilage [47,48]. Expression of *COL2A1* during cartilage development and OA is controversially discussed; both up- and down regulation is reported during early and late OA [22]. However, TNF-α inhibits *COL2A1* expression via transcription factor SOX9 [49], and the expression of *COL2A1* was low in all TNF-α treated micromasses. Interestingly, in contrast to untreated micromasses, cultures that were stimulated with TNF-α and 500 nmol/L CCL25 showed no further significant down-regulation of *COL2A1* in PCR or porcine microarrays. These finding may indicate a cartilage protective effect in OA-like conditions.

Currently, there are limited data available on physiological CCL25 concentrations in serum (range 0.38–5.19 nmol/L) and synovial fluid [50]. In line, one limitation of our study is the unknown CCL25 concentration of FBS. However, micromasses with 0 nmol/L CCL25 but FBS showed no CCL25 effects. Another limitation is that the most effective in vivo CCL25 concentration is unknown. Therefore, a broad range of physiological and supra-physiological concentrations were tested. As seen for other cytokines and growth- and differentiation factors, tested in vitro concentrations are often significantly higher than concentrations found in vivo [51,52]. In this first study with five different CCL25 concentrations combined with or without TNF-α, we focused on cell viability, ECM alterations, and gene expression changes. The duration of cell stimulation with CCL25 should be extended in future experiments for certain concentrations. Another limitation is that the effect of CCL25 on porcine MSC, for example, the induction of differentiation and dedifferentiation, is not further investigated in this study. There is some information dealing with CCR9 and human MSC differentiation in literature [53]. Here, we aimed to focus on the effects of CCL25 on cartilage ECM and resident chondrocytes. An additional drawback is that the dominant effect of TNF-α on tissue-engineered cartilage may superimpose the effect of CCL25 in our study, but we could detect effects when cells were stimulated under normal conditions with CCL25 alone. Another limitation next to the relatively short time of stimulation with CCL25 is the small sample size. However, the aim of this study was to cover a wide range of doses (0–1000 nmol/L CL25) under normal and osteoarthritic conditions in order to identify potential CCL25 associated implications.

## 4. Materials and Methods

### 4.1. MSC and Chondrocyte Isolation

Tissues samples of 15 sacrificed 6–8 months old female pigs (100–125 kg) were obtained from a slaughterhouse. Therefore, no study approval was necessary. Shortly after the pigs were slaughtered, the whole knee joints consisting of femur, meniscus, tibia, and ligaments were packed to protect them from drying out and stored in a cooling box at 4 to 8 °C. They were directly transported to the laboratory. MSC were isolated from femoral bone marrow (*n* = 3 pigs) [54]. Briefly, marrow was diluted in HANK’s solution (Merck, Darmstadt, Germany) and fat was removed. Subsequently, cells were singularized and the suspension was centrifuged at 300× *g* for 6 min and resuspended in DME-medium (Merck) supplemented with 10% fetal bovine serum (FBS; Thermo Fisher Scientific, Dreieich, Germany), 2 ng/mL basic fibroblast growth factor (PeproTech, Hamburg, Germany), 4 mM l-alanyl-glutamine (Merck), 100 U/mL penicillin (Merck), and 100 µg/mL streptomycin (Merck). MSC were plated at a density of 3 × 10^6^/cm^2^, and medium was changed after 72 h. At 90% confluence, MSC were detached with 0.05% trypsin/EDTA and subcultured up to passage 3.

**Figure 6:**
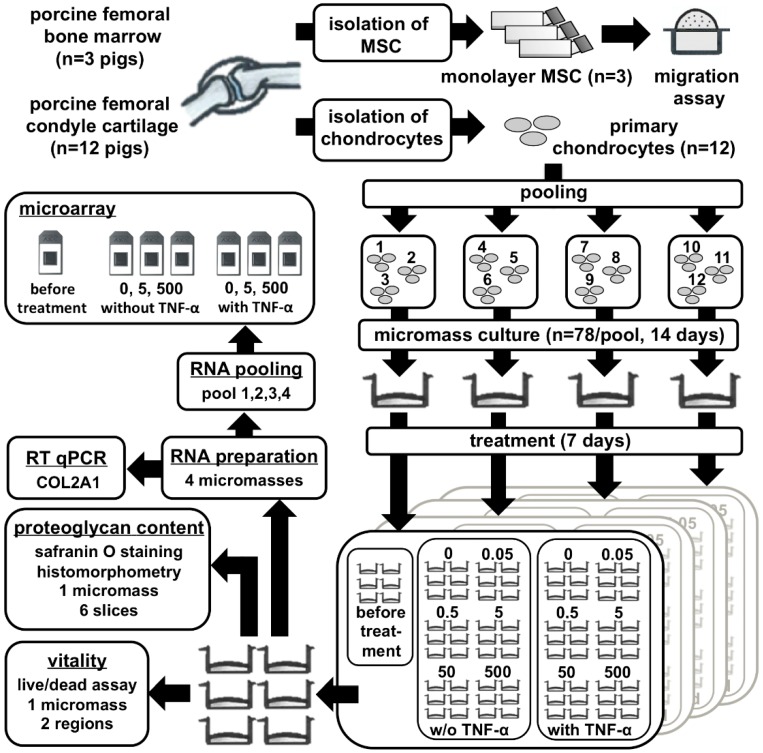
Flow chart of experimental design. MSC from three donors were isolated and subcultured up to passage three before a migration assay was performed with CCL25 (0, 0.1, 1, 10, 100, 250, 500, 1000 nmol/L). Primary chondrocytes from 12 donors were isolated from femoral condyles and pooled equally for micromass cultures (*n* = 4 donor groups). After 14 days, micromasses were stimulated with CCL25 (0, 0.05, 0.5, 5, 50, or 500 nmol/L) with or without (w/o) TNF-α. After seven days of stimulation with CCL25 alone or in combination with TNF-α, two pictures per micromass were analyzed for live/dead assay, six slices per micromass were stained with safranin O for histological analysis, and four micromasses were mechanically homogenized for RNA isolation and analyzed with RT-PCR. Afterwards, RNA from all four donor groups was pooled equally (pool 1,2,3,4) and analyzed with a Porcine Genome Array. The microarray was performed for seven concentrations: micromasses before treatment at day 14, micromasses after seven days of stimulation with 0, 5, 500 nmol/L CCL25 and for micromasses after seven days of stimulation with 0, 5, 500 nmol/L CCL25, and 0.6 nmol/L TNF-α.

Chondrocytes were isolated from femoral condyles (*n* = 12 pigs). Cell isolation was performed as published previously [22]. Briefly, cartilage slices (2–3 mm) were incubated for 19 h at 37 °C in spinner flasks, including an enzymatic solution of RPMI medium (Merck), 10% FBS, 100 U/mL penicillin, 100 µg/mL streptomycin, 333.3 U/mL collagenase II (Merck), 1 U/mL collagenase P (Roche, Mannheim, Germany,) and 33.3 U/mL hyaluronidase (Sigma-Aldrich, Steinheim, Germany). Cells were centrifuged at 400 g for 15 min, resuspended in complete RPMI medium (RPMI with 10% FBS, 100 U/mL penicillin, 100 µg/mL streptomycin, and 170 µM l-ascorbicacid-2-phosphate), and used to generate high-density micromasses. We depicted the entire experiment in Figure 6.

### 4.2. MSC Migration Assay

The migratory effect of recombinant human CCL25 (Order No 300-45; PeproTech) on porcine MSC (*n* = 3 donors) was assessed using 96-well ChemoTx plates with 8 µm pore-size membranes (Neuro Probe, Gaithersburg, MD, USA) [15]. Lower wells contained 40 µL deprivation medium (DMEM, 0.1% FBS) plus different CCL25 concentrations (0, 0.1, 1, 10, 100, 250, 500, 1000 nmol/L). Deprivation medium with 10% FBS served as the positive control. Upper wells were supplied with 3 × 10^4^ MSC per 37.5 µL deprivation medium and incubated for 20 h. Migration assay was performed in triplicates. Migrated cells were fixed with methanol and stained using the Hemacolor Rapid Staining Kit (Merck). Photographs were taken and cell amount per field was enumerated with the Cell Counter plugin of ImageJ software (NIH, Bethesda, MD, USA). Statistical analysis was performed with SigmaStat 3.5 (Systat Software, Erkrath, Germany). Normal distribution of data was checked and one-way analysis of variance (ANOVA) for correlated samples was used with post hoc analysis according to Fishers Least Significant Difference-Test.

### 4.3. Preparation of High-Density Micromass Cultures

To generate tissue-engineered cartilage, chondrocytes were cultivated as 3D high-density micromasses in 96-well plates (Becton Dickinson, Heidelberg, Germany). Each well was filled with 6 × 10^5^ chondrocytes pooled from three out of 12 donors (*n* = 4 donor groups) per 200 µL complete RPMI medium. To achieve ECM build-up, cells were cultivated for 14 days. For the next seven days, medium that was supplemented with 0, 0.05, 0.5, 5, 50, or 500 nmol/L CCL25 was added and changed every 24 h. To simulate OA-like changes, CCL25 treatment was combined with the addition of 0.6 nmol/L TNF-α (R&D Systems, Wiesbaden, Germany) [22]. Micromasses of all four donor groups were used to investigate the effect of 0–500 nmol/L CCL25 on normal (without TNF-α) or OA-like chondrocytes (with TNF-α).

### 4.4. Live/Dead Assay

Chondrocyte micromass cultures (*n* = 4 donor groups) were detached, washed with Phosphate Buffered Saline (Merck), and stained with 0.1 mg/mL propidium iodide for 2 min and 3 µg/mL fluorescein diacetate (both Sigma-Aldrich) for 15 min. Live/dead assay was performed for one micromass per preparation. Two fluorescence pictures were taken and the amount of vital (green) and dead (red) cells was quantified applying the Cell Counter plugin of ImageJ on the green or red color channel, respectively. Statistical analysis was performed with SigmaStat 3.5. ANOVA on ranks, with a subsequent Student-Newman-Keuls-Test was conducted for data of the live/dead assay.

### 4.5. Histological Analysis of GAGs

Chondrocyte micromass cultures (*n* = 4 donor groups) were embedded in Tissue-Tek (Sakura Finetek, Staufen, Germany), snap-frozen in liquid nitrogen, and then cut into 8 µm slices. To display the amounts of GAGs, 0.7% safranin O (Sigma-Aldrich) in 66% ethanolic solution was used, and nuclei were counterstained with 0.2% Fast Green FCF (Sigma-Aldrich). Semi-quantitative histomorphometric analysis to assess the intensity of safranin O staining was performed, as previously published [22]. Briefly, pictures were taken and all pixels in areas of interest were valued in the red-green-blue color mode with a tool based on Xcode (Apple, Sunnyvale, CA, USA). Analysis was done in sextuplicates (*n* = 6 slices per preparation). Statistical analysis was performed, as described for the MSC migration assay.

### 4.6. RNA Isolation and Real-Time RT-PCR

For RNA-isolation chondrocyte micromasses of each experiment (*n* = 4 donor groups) were mechanically homogenized in 1 mL TRI-Reagent (Sigma-Aldrich). 133 µL 1-bromo-3-chloro-propane (Sigma-Aldrich) were added and the solution was centrifuged for 45 min at 13,000× *g*. Subsequently, the upper phase was dissolved in an equal volume of 70% ethanol and filled into a spin column. Further steps were performed as described in the RNeasy Mini Kit protocol (Qiagen, Hilden, Germany). Purity and concentration of RNA was tested using the NanoDrop 1000 spectrophotometer (Thermo Fisher Scientific). RNA 6000 Nano Kit (Agilent Technologies, Santa Clara, CA, USA) was used to test RNA integrity.

For real-time RT-PCR, RNA from chondrocyte micromasses (*n* = 4 donor groups) was reverse transcribed with the iScript cDNA Synthesis Kit (BioRad, Munich, Germany). PCR was performed in triplicates (*n* = 3 wells per preparation) in 96-well plates on the StepOnePlus RT-PCR System (Applied Biosystems, Darmstadt, Germany) using TaqMan probes and primer sets (order no. in parentheses) for *glyceraldehyde-3-phosphate dehydrogenase* (*GAPDH*, Ss03375435_u1) and *collagen type II* (*COL2A1*, Ss03373344_g1). *GAPDH* expression was used to normalize the samples, and *COL2A1* expression is given as a percentage related to *GAPDH* expression. Statistical analysis was performed as described for the MSC migration assay.

### 4.7. Microarray Analysis

RNA of chondrocyte micromass cultures from each of the four donor groups was pooled equally to reduce sample size and used for expression profiling of non-stimulated micromasses at day 14, micromasses treated with 0, 5, or 500 nmol/L CCL25 for seven days (day 14 + 7), and micromasses treated with 0, 5, or 500 nmol/L CCL25 and stimulated with 0.6 nmol/L TNF-α for seven days (day 14 + 7). Analysis was performed with the Porcine Genome Array (Affymetrix, Freiburg, Germany) that contains 23,937 probe-sets representing 20,201 genes.

RNA processing and hybridization were performed in accordance to the manufacturer’s protocol. We used the Affymetrix GeneChip Scanner 3000 supported by GeneChip Operating software 1.4 (GCOS) for readout [55]. Raw data were processed with GCOS and have been deposited in the ArrayExpress database at the European Bioinformatics Institute (EMBL-EBI) under accession number E-MTAB-7430. Briefly, they were normalized and the comparison of all seven GeneChips was performed based on signal, detection, change and fold change (FC) values. Probe-sets that were detected as present, significantly differentially expressed between two groups and FC ≥ 1.5 or FC ≤ −1.5 were further analyzed. Hierarchical clustering was performed with log2-transformed signals normalized by genes and Pearson correlation as distance measure using Genesis 1.8.1 software [56]. Furthermore, genes that were differentially expressed after CCL25 stimulation were imported into DAVID Bioinformatics Resources. Functional annotation charts were generated applying the categories GOTERM_BP_DIRECT (Gene Ontology-Biological Process, GO Consortium) and KEGG_PATHWAY (Kyoto Encyclopedia of Genes and Genomes database) [57,58]. Significance of gene-term enrichment values (EASE score) was estimated using a modified Fisher’s exact test included in DAVID Bioinformatics Resources. *P*-values < 0.05 were considered to be significant. To match names for unnamed porcine probe-set IDs, we used cross-species relationships between porcine and human probe-set IDs (U133PlusVsPorcine_Complex sheet) in combination with the human NetAffx annotation file (Affymetrix HG_U133_Plus_2 Array).

## 5. Conclusions

We demonstrated no negative effects on tissue-engineered cartilage ECM and chondrocyte viability for a dose range of CCL25 (0.05–50 nmol/L). These findings are relevant to establish an in situ repair approach that is based on CCL25 to recruit endogenous repair cells to damaged or degenerated cartilage sites.

## Supplementary Materials

Supplementary materials can be found at http://www.mdpi.com/1422-0067/20/1/52/s1. Table S1: Effect of CCL25 and TNF-α on cartilage marker gene expression, Table S2: Effect of 5 nmol/L and 500 nmol/L CCL25 on tissue-engineered cartilage, Table S3: Effect of 5 nmol/L and 500 nmol/L CCL25 on TNF α stimulated tissue-engineered cartilage.

## Abbreviations

ACAN	aggrecan
ANK3	ankyrin family member ankyrin 3
BGN	biglycan
BSCL2	seipin lipid droplet biogenesis associated
CASQ2	calsequestrin 2
CCL2	C-C motif chemokine ligand 2
CCL25	Thymus-expressed chemokine
CCL3	C-C motif chemokine ligand 3
CCL4	C-C motif chemokine ligand 4
CCL7	C-C motif chemokine ligand 7
CCR9	C-C motif chemokine receptor 9
COL10A1	collagen type X
COL1A1	collagen type I
COL2A1	collagen type II
COMP	cartilage oligomeric matrix protein
CSPG4	chondroitin sulfate proteoglycan 4
CXCL12	stromal cell-derived factor 1α
CXCL2	C-X-C motif chemokine 2
DCN	decorin
ECM	extracellular matrix
EGR1	early growth response protein 1
ESPN	espin
ETS1	transcription factors like ETS proto-oncogene 1
FMOD	fibromodulin
FOSB	FosB proto-oncogene, AP-1 transcription factor subunit
GAD2	glutamate decarboxylase 2
GAPDH	glyceraldehyde-3-phosphate dehydrogenase
HAPLN1	link protein
IL18	interleukin 18
IL-1β	interleukin 1 beta
IL8	interleukin-8
IVL	involucrin
JAK2	janus kinase 2
JUN	jun proto-oncogene, AP-1 transcription factor unit
JUNB	jun B proto-oncogene
LEPREL4	leprecan-like 4
LUM	lumican
MAPK	mitogen-activated protein kinases
MARVELD3	tight-junction component MARVEL domain-containing protein 3
MMP	matrix metallopeptidases
MSC	mesenchymal stem- or stromal cells
OA	osteoarthritis
PI3K-Akt	phosphatidylinositol 3-kinase- serine/threonine kinase
POSTN	periostin
PTGES	prostaglandin E synthase
SLC12A1	solute carrier family 12 member 1
SMPD1	sphingomyelin phosphodiesterase 1
SOD2	superoxide dismutase 2
SOX9	SRY-box 9
TNF-α	tumor necrosis factor-α
TTC28	tetratricopeptide repeat domain 28
VCAN	versican
